# Clinical Study and Serological Diagnosis of Vector-Borne Pathogens in Sardinian Dogs

**DOI:** 10.3390/vetsci11070313

**Published:** 2024-07-12

**Authors:** Valentina Chisu, Antonio Tanda, Sara Sechi, Maria Luisa Pinna Parpaglia, Gabriella Masu, Federica Loi, Giovanna Masala

**Affiliations:** 1 Istituto Zooprofilattico Sperimentale “G. Pegreffi” della Sardegna, Via Duca degli Abruzzi 8, 07100 Sassari, Italy; valentina.chisu@izs-sardegna.it (V.C.); antonio.tanda@izs-sardegna.it (A.T.); gabriella.masu@izs-sardegna.it (G.M.); giovanna.masala@izs-sardegna.it (G.M.); 2 Teaching Veterinary Hospital, University of Sassari, Via Vienna 2, 07100 Sassari, Italy; sarasechilavoro@tiscali.it (S.S.); pinnapar@uniss.it (M.L.P.P.); 3 Osservatorio Epidemiologico Veterinario Regionale della Sardegna, Istituto Zooprofilattico Sperimentale della Sardegna, 07100 Sassari, Italy

**Keywords:** dogs, ticks, tick-borne diseases, multiple correspondence analysis, pilot study

## Abstract

**Simple Summary:**

In regard to Sardinia, studies on canine vector-borne diseases are scarce. This study aimed to examine the most common vector-borne diseases affecting domestic dogs in this area and how clinical signs are associated with the disease. The results highlight that 48% of the tested dogs were positive for at least one pathogen, with 34.5% of them having significant clinical symptoms resembling vector-borne disease infections and 65.5% having no signs. These results suggest that preventive measures should be taken to control the spread of vectors and to reduce the infection risk to humans and pets.

**Abstract:**

Canine vector-borne diseases (CVBDs) comprise a group of infectious diseases caused by a wide range of pathogens transmitted by arthropod vectors. Clinical signs commonly involve symptoms such as fever, anorexia, weight loss, blood disorders, hepatosplenomegaly, and others that can lead to death in dogs with comorbidities. Some pathogens responsible for CVBDs constitute a serious threat to human health due to their zoonotic transmission. This study aimed to determine the prevalence of zoonotic vector-borne diseases (*Rickettsia rickettsii*, *Anaplasma phagocytophilum*, *Ehrlichia canis*, *Bartonella henselae*, and *Leishmania infantum*) in domestic Sardinian dogs with and without clinical signs of these pathogens. Blood serum samples were collected from 142 dogs and examined through serological analysis. Clinical signs suggestive of these pathogens were also evaluated. The results obtained showed that 33 (33/140; 23.6%), 22 (22/134; 16.4%), 14 (14/142; 9.9%), 20 (20/66; 30.3%), and 26 (26/108; 24.1%) dogs were seropositive for *Rickettsia* sp., *Anaplasma* sp., *Ehrlichia* sp., *Bartonella* sp., and *Leishmania* sp. antibodies, respectively. Among these dogs, 12 dogs presented with at least one clinical sign (8.5%), while 18 (12.7%) showed more than two symptoms at the same time. Furthermore, among the asymptomatic dogs (93/142; 65.5%), 13% (*n* = 12) tested positive for *A. phagocytophilum*, 12% (*n* = 11) tested positive for *B. henselae*, 9% (*n* = 8) tested positive for *E. canis*, 12% (*n* = 11) tested positive for *L. infantum*, and 19% (*n* = 18) tested positive for *R. rickettsii*. This survey represents the first study assessing different canine vector-borne pathogens in dogs from North Sardinia. Since the pathogens detected here represent emerging zoonotic diseases, these results highlight the need to undertake further studies to increase the knowledge of these under-reported vector-borne pathogens in Sardinia.

## 1. Introduction

Canine vector-borne diseases (CVBDs) represent an important group of diseases that affect dogs worldwide [1]. Even though CVBDs are highly endemic in tropical and subtropical regions, an abundance of novel vector-borne diseases (VBDs) has recently been identified in the canine population in temperate regions [2]. Biting arthropods, including ticks, fleas, lice, triatomines, mosquitoes, tabanids, and phlebotomine, are known to be important vectors in transmitting a wide range of pathogens to dogs [3]. In past decades, humans have formed closer relationships with pets at all latitudes and in all socioeconomic contexts [4]. However, these behaviors can increase pet-associated zoonosis risks [5]. Moreover, VBDs are related to several factors such as climatic and ecologic changes, the availability of improved diagnostic tools, and increases in animal movements [6,7]. In particular, climate changes can modify the distribution of vectors and their life cycle, survival, and reproduction [5]. 

In such a context, the control of CVBDs is essential both for the health of dogs and public health [8]. The transmission of pathogens can only be avoided by ectoparasite treatment of owned and stray dogs. If not adequately treated with ectoparasites, these dogs could become a reservoir for pathogens [9], representing an important risk factor for the transmission of VBDs in areas where the vector density is high. This is the case for pathogens such as *Leishmania infantum*, the causative agent of infantile visceral leishmaniasis in the Mediterranean region and Latin America [10]. Its spread is increasing in geographic areas previously defined as non-endemic due to a combination of biological and environmental factors (e.g., climate, holiday travel of dogs with their owners to endemic areas, and adoptions from the south of Europe toward central and northern Europe) [11]. Furthermore, other species in the Rickettsiales order are the cause of well-known infectious agents reported in small animals and humans worldwide [12]. 

Canine VBDs are often characterized by subclinical infection or are reported in association with generic clinical signs (i.e., fever, lethargy, weight loss, lymphadenomegaly, and mild lameness). Frequently, these clinical signs overlap with those of other diseases [13,14,15], or symptoms are vague and nonspecific, and for this reason, they are commonly under-reported. Dogs that are subclinically infected can be reservoir hosts for arthropod-transmitted zoonotic pathogens [16], and sometimes the VBD can have severe health implications such as thrombocytopenia and/or death in Ehrlichia and Anaplasma infections [17,18]. Clinical signs detected in infected dogs caused by *Ehrlichia canis*, a Gram-negative obligate intracellular bacterium, may persist for many years, and the disease usually becomes chronic [19].

The need for enhancing VBD surveillance and educational and risk-communication programs on personal protection against vectors is increasing worldwide, given that peri-urban areas are being ever-increasingly affected by vector expansion [20].

Otherwise, the control of these VBDs is often compromised by limited knowledge of their biology, epidemiology, and the mechanism of interaction. The Mediterranean basin represents a suitable environment for monitoring the local canine population and updating the epidemiological data on VBDs in this area. Despite their recognized importance, many aspects concerning the epidemiology and public health significance of CVBDs in Sardinia are still poorly known, and the data have not been comprehensively discussed. In Sardinia, important parasitic and bacterial vector-borne diseases have been previously confirmed in ticks and domestic ruminants by molecular studies [21,22,23]. However, to date, little is known about the seroprevalence of these pathogens in Italian dogs [14]—particularly on the main island of Sardinia [24,25]—or whether these important tick-borne diseases could have the potential to determine clinical symptoms in infected dogs. Considering the lack of evidence-based information on these pathogens and associated clinical signs, the aim of this voluntary-based study was to serologically screen Sardinian dogs to preliminarily investigate the potential role that these pathogens have in the clinical features of VBDs.

## 2. Materials and Methods

### 2.1. Ethical Statement

All methods were carried out in accordance with relevant guidelines and regulations. The study did not involve any animal experiments. Sample collection was carried out during the routine clinical visits of dogs at the Teaching Veterinary Hospital (TVH) of the University of Sassari (Sardinia–North area).

### 2.2. Study Design and Sample Collection

During the study plan, hypothetical sample size calculations were performed. At least 140 dogs needed be recruited and tested for each VBD to detect a minimum prevalence of 10%, starting from the 20% baseline prevalence calculated by the historical data [21,22,23], with a power of 80% (α—0.05, two-sided). Considering the retrospective nature of this study, all samples routinely collected by the TVH between September 2020 and December 2021, and meeting the inclusion/exclusion criteria, were included. The inclusion criteria were: (i) being tested for at least one pathogen, (ii) the presence of informed consent signed by the dog’s owner, (iii) availability of epidemiological information, and (iv) no more than 10% missing data. Considering that the dogs involved were tested based on clinical suspicion, not all dogs were tested for every pathogen. Epidemiological data on the dog (i.e., breed, sex, age at presentation, and features related to VBD prevention) were recorded by the facility staff, with the help of the dog’s owner, at the time of presentation. Retrieval of epidemiological missing information was not possible, neither was performing further laboratory analyses on blood samples.

Upon clinical examination, peripheral blood samples (total of 5 mL) were collected by cephalic or jugular venipuncture by trained project staff. The presence of veterinarians was ensured for supervision during all sampling practices. Blood samples were collected into a 10 mL vacuum tube, refrigerated, and transported to the laboratories of the Istituto Zooprofilattico Sperimentale della Sardegna. The serum was obtained from each sample by centrifugation before subsequently being stored at −20 °C while awaiting antibody testing. 

### 2.3. Immunofluorescence Test

All serological analyses were conducted within the laboratories of the Istituto Zooprofilattico Sperimentale of Sassari, Sardinia, Italy.

All sera were analyzed using an Immunofluorescent Antibody Test (IFAT) to determine the presence of circulating IgG antibodies against *Rickettsia rickettsii*, *Anaplasma phagocytophilum*, *Ehrlichia canis*, *Bartonella henselae*, and *Leishmania infantum*. 

Anti-*Rickettsia* spp. antibodies were detected using a commercial canine *Rickettsia rickettsii* IgG IFA KIT (IFA Substrate slide, Fuller Laboratories, Fullerton, CA, USA), according to the manufacturer’s guidelines. Given that antibody titers are generally associated with apparent clinical signs, and mainly at the first sample collection, they are potentially related to nonspecific fluorescence. A titer of at least 1:128 was considered positive and was selected to eliminate low antibody titers.

Slides containing *A. phagocytophilum* antigen (VMRD Laboratories, Pullman, WA, USA) were used for detecting antibodies against *A. phagocytophilum*. A titer of at least 1:40 was considered positive. 

Anti-*E. canis* antibodies were detected by IFA using slides containing fixed *E. canis* antigen (ATCC no. CRL10390) used for antigen preparation in monocyte–macrophage cells (DH82), as described by Masu et al., 2012 [25]. Infected cultures were split onto DH82 cells grown in Earle’s salts Minimal Essential Medium (E/MEM, Gibco—Life Technologies Corporation, New York, NY, USA), supplemented with 2 mM l-glutamine (200 mM) and 15% (*v*/*v*) fetal calf serum (FCS, Gibco—Life Technologies Corporation, New York, NY, USA) at 37 °C with 5% CO_2_. After about a month, *E. canis*-infected DH82 cultures were harvested by centrifugation at 800× *g* for 10 min and washed twice with phosphate-buffered saline (PBS, 0.1 M phosphate, 0.33 M NaCl, pH 7.2). The pellets were suspended in PBS and dropped (10 μL) onto each well of 12-well teflon-coated slides (Immuno-Cell Int., Mechelen, Belgium). Slides were air-dried, fixed with cold acetone, and stored at −20 °C until use. A titer of at least 1:80 was considered positive. 

A commercial IFAT diagnostic test (Agrolabo, Scarmagno, TO, Italy) was used for *B. henselae* antibody detection, according to the manufacturer’s instructions. Samples were scored as positive when they produced a clear cytoplasmic and membrane fluorescence from a cut-off dilution of 1:40. 

For *L. infantum* diagnosis, IgG antibodies were detected in serum samples by IFAT according to the Italian National Reference Centre for Rickettsiosis (CRABaRT—Istituto Zooprofilattico Palermo, Italy). Dog serum was diluted by 1:40, 1:80, and 1:160 and screened for the qualitative detection of IgG antibodies for *Lh. infantum.* A titer of at least 1:160 was considered as positive, while titers of 1:40 and 1:80 were considered doubtful.

Positive sera were titrated until negative results were obtained. The highest dilution showing fluorescent promastigotes was taken to be the antibody titer, whereas samples solely showing fluorescence at a 1:40 dilution were considered exposed but not infected.

The positive and negative serum were included as positive and negative controls in each test. The results were visualized using standard fluorescence microscopy, where a positive reaction was seen as sharply defined apple-green as well as being absent in the negative control.

### 2.4. Statistical Analysis

All collected data were stored in an electronic database using a closed-response data collection instrument (Microsoft Excel, Microsoft Corporation, Redmond, WA, USA), and were password protected. Before proceeding with the statistical analysis, data quality and completeness were tested, and any ill-formed variables were identified and corrected with the support of the facility staff. 

Descriptive analyses were performed based on number and percentage, to evaluate the variables’ distribution at baseline. Considering the non-inferential purpose of this work, and its aim of proposing a possible CVBD profile, multiple correspondence analysis (MCA) was applied. The use of MCA is particularly relevant in studies where a large amount of qualitative data is collected, often paired with quantitative data. 

MCA graphical representations help to simplify the process of interpreting the relationships among several categorical dependent variables [26]. Their graphical display is used to summarize the proximities between the subjects and to provide a structural organization for the variables and categories in a dimensional space that is useful for identifying patterns between dummy variables [27]. Points (categories) that are close to the mean are plotted near the MCA plot’s origin and those that are more distant are plotted farther away. Categories with a similar distribution are near one to another in the map as clusters, while those with different distributions stay farther apart. In a two-dimensional graphical display of the data, categories sharing similar characteristics are located close together, forming point clouds. 

MCA is performed on an *I* × *Q* indicator matrix, in which *I* is the set of *i* individual records (dogs) and *Q* is the set of categories of all variables. Thus, the component in the cell (*i*, *q*) consists of the individual record *i* and category *j*. Associated categories in MCA are placed close together in a Euclidean space, leading clouds, or a combination of points that have similar distributions. Notably, MCA produces point clouds, which are usually defined by two dimensional graphs.

Data on VBDs (*R. rickettsii*, *A. phagocytophylum*, *E. canis*, *B. henselae*, *L. infantum*) were used as active variables in the MCA. Age category, sex, and clinical signs were used as supplementary variables that do not contribute to the MCA, but can be plotted together to provide useful visualization of the distribution of the features along the development gradients and to validate the components [28]. Variables were selected using the square cosine test [29], and all those with cos2 > 0.2 in at least one dimension were maintained.

To retain the maximum number of dimensions, those with inertia above 0.2 [30] were retained and included in the final MCA. First, overall MCA was performed, including all VBD outcomes (*R. rickettsii*, *A. phagocytophylum*, *E. canis*, *B. henselae*, *L. infantum*) and all explicative variables [31]. All statistical analyses were performed using the Factoshiny [31] package in R software version 4.3.3 (R Development Core Team, 2015).

## 3. Results

A total of 186 dogs were included in this study. After inclusion/exclusion criteria evaluation, 44 dogs were excluded and 142 serum samples from owned dogs, collected between September 2020 and December 2021 (Table 1), were used for the analysis. All 142 samples included were tested for *E. canis*, but only 66 were tested for each pathogen. This sample size was less than the sample needed to provide generalizable results (i.e., representative sample); thus, a non-inferential statistical approach was applied.

A total of 68 (48%) dogs tested positive for at least one pathogen: 22 (16.4%) dogs tested positive for *A. phagocytophylum*, 20 (30.3%) tested positive for *B. henselae*, 14 (9.9%) tested positive for *E. canis*, 26 (24.1) tested positive for *L. infantum,* and 33 (23.6%) tested positive for *R. rickettsii* circulating antigens. Table 1 shows the distribution of seropositivity for each pathogen by age groups, sex, breed, and symptoms. Given the planned statistical analyses, the age feature was categorized as young (<5 years old; 100 dogs), adult (5–10 years old; 33 dogs), and old (>9 years old; 9 dogs). A total of 56, 16, and 6 seropositive dogs were detected in young, adult, and old dogs, respectively. The overall sample was well-balanced for sex (47% female, 52% male) and no statistically significant differences were detected in the presence of vector-borne pathogens between sex categories (85% positive female, 77% positive male). Mixed breed dogs (including the owned dogs and those from the kennels) were the most common breed (*n* = 85), followed by the Cirneco of Etna breed (*n* = 32).

Based on clinical signs, dogs were classified as symptomatic if at least one sign of illness was reported, or asymptomatic otherwise. Symptoms included general clinical signs, as illustrated in Table 1. Specifically, 93 (65.5%) dogs had no clinical signs (asymptomatic); among them, 12 (12.9%) tested positive for *A. phagocytophilum*, 11 (11.8%) tested positive for *B. henselae*, 8 (8.6%) tested positive for *E. canis*, 11 (11.8%) tested positive for *L. infantum*, and 18 (19.3%) tested positive for R. rickettsii. 

Among the symptomatic dogs, the most common symptom was weight loss (33, 67%), cutaneous symptoms (25, 51%), adenomegalia (20, 41%), difficulties with walking (18, 37%), tenderness (17, 35%), edema (15, 31%), and depression and lameness (14, 29%). No dog presented with fever.

Among all the dogs involved in this study, most of them did not undergo antiparasitic treatments (92, 64.8%), 109 did not recover in a doghouse (76.8%), and 94 came from a kennel (66.2%). Ticks were found in 41 (28.9) of the sampled dogs, particularly in 31 (33.7%) of the dogs without antiparasitic treatment, and in 34 (31.2) dogs that had not recovered in a doghouse. A strong positive correlation (correlation coeff. = 0.77, *p*-value < 0.0001) was found between prevention factors (antiparasitic treatment and living indoors); among those who underwent antiparasitic treatment (*n* = 50), 33 (66%) recovered in a doghouse, while no dogs without antiparasitic treatment recovered in a doghouse (*n* = 92). On the contrary, a negative correlation (correlation coeff. = −0.94, *p*-value < 0.0001) was found for prevention factors and staying in a kennel; only three dogs from a kennel underwent antiparasitic treatment and none recovered in a doghouse. 

**Figure 1:**
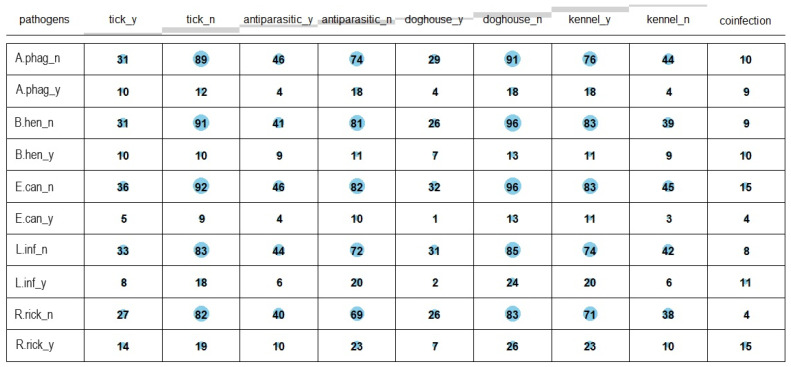
Bubble correlation matrix between pathogen positivities and prevention variables.

A bubble correlation matrix between pathogen positivities and prevention variables is reported in Figure 1. Moreover, 19 (13.4%) dogs showed seropositivity for two or more pathogens. Detailed results for coinfections are listed in Table 2.

### Multiple Correspondence Analysis

MCA was applied to the collected data. From the MCA analysis, a three-dimension MCA solution was considered the most adequate. As reported in Table 3, the model resulted in three dimensions, explaining 55.32% of the variance. 

The dimensions presented are, respectively, eigenvalue—2.961, 1.430, and 1.141; inertia—0.283, 0.165, and 0.126; and Cronbach’s alpha—0.811 (95% CI 0.705–0.926), 0.788 (95% CI 0.583–0.852), and 0.696 (95% CI 0.665–0.735).

**Figure 2:**
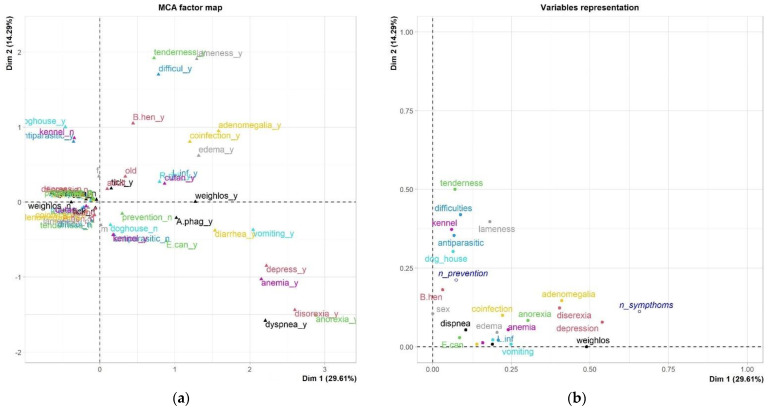

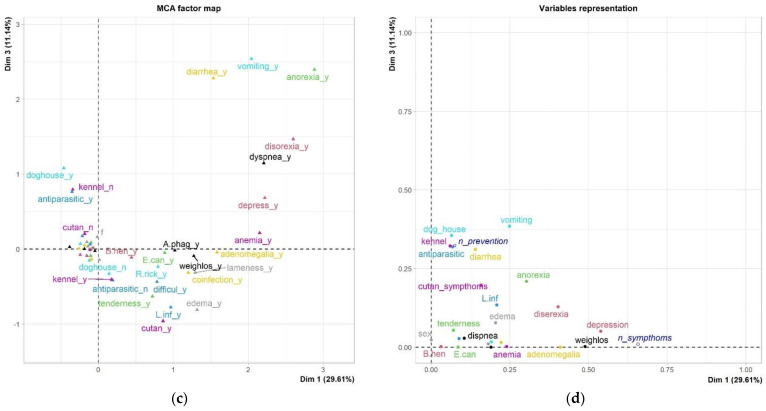
MCA plots. (**a**) MCA factor map between dimension 1–2; (**b**) Variable representations between dimension 1–2; (**c**) MCA factor map between dimension 1–3; (**d**) Variable representations between dimension 1–3. The plot gives a correlation measure between variables and each dimension: the distance between any row points or column points gives a measure of their similarity (or dissimilarity); thus, points with similar profiles are close on the factor map.

Figure 2 illustrates the MCA plot and variable representation. The MCA plot shows the distribution of all category coordinates. This plot gives us an idea of the variable categories’ positions in the three-dimensional space, based on their eigenvalues. 

The MCA plots below are from a table consisting of 142 rows, each representing a different dog, and 30 columns. The origin of the two axes is where the x- and y-axes are both at zero, and it is shown as the intersection of two dashed lines. The farther labels are from the origin, the more discriminating they are. Discrimination measures (Table 4 and Figure 2a,c) and a joint plot of category points were obtained (Figure 2b,d).

There were clear differentiating values allocated to each of the obtained dimensions (Table 2). The maximum value of the discrimination measures was 8.09 (disorexia) for the 1st dimension, 12.84 (tenderness) for the 2nd dimension, and 13.58 (vomiting) for the 3rd dimension. Gender slowly contributed to the eigenvalue of the second dimension (value 1.62 and 1.45), while age class was not differentiated based on any of the data in the study (contribution value < 1.0). The most discriminant variables for Dimension 1 hierarchically were disorexia, weight loss, adenomegalia, and anorexia; regarding Dimension 2, the most discriminant variables were tenderness, difficulties with walking, lameness, depression. Finally, the most discriminant variables for Dimension 3 hierarchically were vomiting, diarrhea, recovering in a doghouse, and antiparasitic treatment (Table 4 and Figure 1). Most of the variables related to pathogen positivities and coinfection presented relevant and similar discrimination measures in the 1st dimension—except for *B. henselae* and *L. infantum*, which mainly contributed to Dimension 2 and 3, respectively. From the results and their graphical visualization, Dimension 1 was termed “Pathogen positivity and general symptoms”, the second dimension “psychological symptoms”, and the third dimension “preventive measures”. 

Only correlations above 0.30 were considered to have meaningful practical significance. In Dimension 1, presence of coinfection significantly correlated with adenomegalia (*r* = 0.356, *p*-value = 0.0021) and edema (*r* = 0.368, *p*-value = 0.0013). *A. phagocytophilum* and *E. canis* correlated with disorexia (*r* = 0.312 and *r* = 0.328, respectively; *p*-values < 0.0001). *R. rickettsii* significantly correlated with adenomegalia (*r* = 0.301, *p*-value = 0.0003) and edema (*r* = 0.327, *p*-value = 0.0043). In Dimension 2, *B. henselae* significantly correlated with tenderness (*r* = 0.400, *p*-value = 0.0141) and difficulties with walking (*r* = 0.353, *p*-value = 0.041). In Dimension 3, a significantly positive correlation was found between *L. infantum* positivity and cutaneous symptoms (0.542, *p*-value < 0.0001), and *L. infantum* positivity and the kennel origin (*r* = 0.416, *p*-value = 0.035). Otherwise, *L. infantum* significantly negatively correlated with antiparasitic treatment (*r* = −0.395, *p*-value = 0.0003) and recovery in a doghouse (*r* = −0.424, *p*-value = 0.019). 

## 4. Discussion

The presence of VBD pathogens has been described in a few studies carried out in Italy [17,23,24], but epidemiological information concerning seroprevalence remains limited. In this study, the occurrence of antibodies against selected causative agents of CVBDs (*R. rickettsii*, *A. phagocytophilum*, *E. canis*, *B. henselae*, and *L. infantum*) associated with clinical features was evaluated among asymptomatic and symptomatic dogs from Sardinia (Italy). Exposure to at least one of five tested pathogens was detected in 48% (*n* = 68) of the 142 tested dogs, while multiple exposure was detected in 21% (*n* = 31) of the dogs involved. 

The highest prevalence (23.6%) among the tested pathogens was detected to *Rickettsial* infection with antibodies against *R. rickettsii*. These results agree with previous studies in which the prevalence of *Rickettsia* in dog serum ranged from 15.5 to 74%, depending on to the geographical area, and the highest values were observed in southern Italy [32]. The IFI test here used for detecting *Rickettsial* antibodies was used as the reference assay. This test is currently considered the gold standard in the serological diagnosis of rickettsiosis, both in human and veterinary medicine [33]. However, it is not specific due to the cross reactions demonstrated among *Rickettsia* spp., and it is not always able to distinguish between the various SFG *Rickettsia* infections [34]. Thus, the seroprevalence could be overestimated due to serological cross-reactivity with other SFG Rickettsia species, as well as those already detected in Sardinia [21,35]; however, *R. rickettsii* seropositivity in dogs from this study is most likely not really related to this microorganism, as this species is not currently present in Europe [12]. The presence of several members of *Rickettsiaceae* has been reported in ticks from Sardinia, mainly in *Rhipicephalus sanguineus* ticks, the most common tick species infecting dogs that is widely distributed on the island [36]. Due to the high distribution of *Rh. sanguineus* (s.l.), the studied area is endemic for Mediterranean spotted fever, with an incidence of 10/10,000 inhabitants per year [36]. The clinical picture of positive dogs resulting from the MCA analysis was characterized by adenomegaly, weight loss, anorexia, lameness, and difficulties with walking, reflecting what is already reported for this infection [23]. It has been known that *Rickettsia* infection induces a sub-clinical form in dogs, characterized by a few days of bacteremia and a slight increase in temperature, lymphadenopathy, anorexia, lethargy that may develop to anemia, and liver, pulmonary, renal, or cerebral dysfunction [23].

The high proportion of *L. infantum* antibodies (24.1%) in the sampled dogs agrees with the endemic status of Sardinia for *leishmaniasis*, in place since the 1990s [37]. More than 250 human cases were reported between 1922 and 2014 [22]. Furthermore, the presence in the island of *Phlebotomus perfiliewi* and *P. perniciosus*, which are proven vectors of the biological transmission of *Leishmania*, was confirmed several times [38]. The MCA results showed significant associations between *L. infantum* antibodies presence and prevention factors when describing the 2nd dimension; 24.1% of the seropositive dogs showed clinical signs consistent with canine leishmaniasis (canL), as well as cutaneous lesions (57.7%) and adenomegaly (42.3%). It was interesting to observe that all symptomatic dogs affected by *L. infantum* infection showed cutaneous lesions (*n* = 15, 100%). On the contrary, 54.4% of seropositive dogs did not show any symptoms, indicating a subclinical infection that could represent a transitory condition [32]. Otherwise, these associations could be affected by the confounding factors described above (i.e., testing by suspicion, an unbalanced sample). Indeed, the presence of coinfection could lead to the progression of the clinical diseases [22]. 

In this study, we confirmed the presence of *A. phagocytophilum* in Sardinian dogs, with 16.4% of the dogs tested being infected, indicating an important circulation of the bacterium in this region. In Italy, *A. phagocytophilum* is a common TBP in domestic dogs, with a reported seroprevalence as high as 38% [1]. The most common symptoms detected in *A. phagocytophilum*-infected dogs were weight loss (41%), adenomegaly (in 31.8% of cases), depression (27.3%), and cutaneous symptoms (27.3%). Less commonly, vomiting and diarrhea, reluctance to move (13.6%), and anorexia (13.6%) also occurred. Skin lesions and vascular lesions associated with moderate–severe edema and erythrocyte extravasation have been observed in dogs with anaplasmosis [38]. However, 12 positive dogs did not show symptoms, in accordance with other studies highlighting the event of asymptomatic infection, and the serological prevalence in animals’ inhabitant endemic areas was not accompanied by clinical findings [39]. Although *A. phaocytophilum* is primarily transmitted by ticks of the genus *Ixodes* in the USA (*I. scapulari, I. pacificus*), Europe (*I. ricinus*), and Asia (*I. persulcatus*), other tick species are also reported to play a role in its epidemiological cycle. In Sardinia, some studies have shown *A. phagocytophilum* in ticks belonging to the *Rhipicephalus and Hyalomma genus* [21]. We can hypothesize the involvement of these tick species in transmission through their bites, but further studies should be performed to better understand the role of these ectoparasites. 

Serological analysis based on the sampling from this study highlights that the *E. canis* seroprevalence was lower (9.9%) than the seroprevalence previously reported in Sardinian dogs (62.5%) [24]. The circulation of tick vectors plays a crucial role in the spread of the etiological agent of canine monocytic ehrlichiosis (CME). Observations from a Sardinian study described the presence of *E. canis* in *Rhipicephalus bursa* tick species [40], although the vectorial competence of these vectors has not yet been evaluated. The disease is typically characterized by fever, depression, anorexia lymphoadenomegaly, splenomegaly, hemorrhagic tendencies, pale mucosa, weight loss, ophthalmologic lesions, and neurologic disorders [41]. Clinical signs may vary due to strain pathogenicity, the dose of the infecting organism, the immunological status of the host, and co-infection with other tick-borne diseases. In this study, infected dogs showed common symptoms of CME, but no statistical significant correlation with the factors used in this investigation was detected. 

Furthermore, this study reveals a high prevalence rate of *Bartonella-henselae*, in 30.3% of the tested dogs. These data agree with those published by Zobba et al. (2007) [42], in which a *B. henselae* seroprevalence of *28% (58/205)* was reported *in* the Sassari district. Since dogs represent an important reservoir for human bartonellosis [43], the effective control of canine-originated diseases is an extremely challenging task. In dogs, a wide variety of *Bartonella* sp. clinical signs has been associated with splenomegaly, nasal discharge, epistaxis, and lameness [44]. According to these clinical abnormalities, the signs of seropositive dogs include adenomegaly, difficulties with walking, lameness, and tenderness. The higher rate of *B. henselae* infections is concerning, since it is considered an important cause of long term intra-erythrocytic bacteremia in both humans and animals. 

Besides the VBD prevalence estimation, several associated symptoms were described. The most common symptoms reported in symptomatic dogs were weight loss (mainly associated with *L. infantum, R. rickettsii, A. phagocytophilum*, and *E. canis* infection), cutaneous symptoms (mainly associated with *L. infantum, R. rickettsii*, and *E. canis* infection), adenomegalia (mainly associated with *L. infantum, R. rickettsii*, and *A. phagocytophilum* infection), difficulties with walking (mainly associated with *Bartonella-henselae* infection), tenderness (mainly associated with *Bartonella-henselae* infection), edema, depression (mainly associated with *E. canis* infection), disorexia (mainly associated with *E. canis* infection), and lameness. 

Moreover, the incidence of VBDs in dogs living outdoors was higher than that for those living in a house, which indicates that the former are at a greater risk of exposure to ticks of different general types that act as vectors for pathogens. In total, 64.8% of the dogs did not receive any antiparasitic treatment, while 35.2% received ectoparasicides, as stated by the dogs’ owners in this study. This result is interesting, since it indicates that dogs are highly exposed to VBDs despite the use of ectoparasicides. We can postulate that these positivities are related to the irregular use of specific treatments—thus increasing the risk of ectoparasite infestations and the transmission of diseases, and favoring the presence of infected arthropods in domestic environments and households. Important differences were found in kennel dogs, which represented 66.2% of the analyzed dogs and which were found to be infected more often than owned dogs (33.8%). However, the kennels from this study were in peri-urban areas where the dogs lived outdoors during the day, and this increased the risk of infection.

Even though most of the positive dogs were young (*n* = 56, prevalence = 56%), seroprevalence seemed to increase with the dogs’ age (old positive dogs *n* = 6, prevalence = 66%). However, considering the unbalanced sample, this association is not statistically significant. 

Several limitations of this study, which also affected the statistical inference, were represented by its retrospective nature, the absence of a study design, and the origin of the samples. The sample involved was unbalanced for several baseline features, as well as age and prevention factors. This hinders the possibility of estimating risk measures. Furthermore, the dogs involved were tested based on clinical suspicion, and this could represent a large confounding factor for the features collected and the VBD antibody detection. This results in a possible overestimation of the seroprevalence, as well as for the frequency of clinical symptoms. Indeed, the samples included were not representative of the overall Sardinian dog population, but only for the North area (Sassari province).

Otherwise, the samples involved allowed the carrying out of an explorative study and the performance of a component analysis with a good level of accounted variance (55%), which will be of great help in planning future studies aimed at in-depth evaluations of the VDB seroprevalence in Sardinia. After all, the seroprevalence estimation reported mainly agrees with previous studies, and differences between epidemiological studies should be attributed to the diagnostic test used, the geographical area, the population chosen, the sampling methods, and the existence of reservoir species, and not solely to the different diffusions of these pathogens [14].

## 5. Conclusions

This study confirms the endemicity of *Leishmania infantum* and *Rickettsia* spp. in Sardinia. Additionally, other CVBDs were detected in dogs from this study, and this indicates that ticks and sand flies in Sardinia are vectors for a large variety of human and zoonotic pathogens.

Dogs, especially those that live or frequent environments largely infested with ticks, are at risk of infection with “tick-borne” agents, but further investigations are necessary to define the real pathogenic role that these latter microorganisms may have towards such animals. Clinical veterinarians who suspect that they are dealing with a case of CVBD should request serological tests for the screening of antibodies against the main vector-borne diseases circulating in the study area. Clinics should know the current epidemiological situation of VBDs. Furthermore, clinicians should request, in addition to serological tests, molecular analyses that could provide more data to formulate a correct diagnosis; furthermore, where possible, sequencing would allow for the exact definition of the etiological agent.

Finally, although VBDs remain quite rare diseases in Italy, considering that peri-urban areas are ever-increasingly affected by vector expansion, in the light of the increasing frequentation of urban and peri-urban green spaces, the results obtained highlight the need of enhancing surveillance, educational, and risk-communication programs on personal protection [20] against VBDs.

## Figures and Tables

**Table 1:** Baseline distribution of the overall sample and the vector-borne pathogens detected, by age, sex, breed, and symptoms. Data are presented as number (percentage, %), and 95% confidence intervals (95% CI) were included for prevalence, reported as the number of positive dogs.

Epidemiological Data	Total Dogs	*A. phagocytophilum* (*n*, %)	*B. henselae* (*n*, %)	*E. canis* (*n*, %)	*L. infantum* (*n*, %)	*R. rickettsii* (*n*, %)
**Tested dogs**	142	134	66	142	108	140
**Number of positive dogs**	68 (47.8; 38.7–55.8) ^¥^	22 (16.4; 10.6–23.8)	20 (30.3; 19.6–42.8)	14 (9.9; 5.5–16.0)	26 (24.1; 16.3–33.2)	33 (23.6; 16.8–31.5)
**Age**						
Young (≤5 years-old)	100 (70.4)	15 (68.2)	12 (60.0)	8 (57.1)	16 (61.5)	22 (66.7)
Adult (5–10-years-old)	33 (23.3)	3 (13.6)	6 (30.0)	5 (35.7)	7 (27.0)	8 (24.2)
Old (≥ 10 years-old)	9 (6.3)	4 (18.2)	2 (10.0)	1 (7.1)	3 (11.5)	3 (9.1)
**Gender**						
Female	67 (47.2)	11 (50.0)	11 (55.0)	7 (50.0)	14 (53.8)	14 (42.4)
Male	75 (52.8)	11 (50.0)	9 (45.0)	7 (50.0)	12 (46.2)	19 (57.6)
**Breed**						
American staffordshire terrier	2 (1.4)	0 (0.0)	0 (0.0)	0 (0.0)	0 (0.0)	0 (0.0)
Border collie	1 (0.7)	0 (0.0)	0 (0.0)	0 (0.0)	1 (3.8)	0 (0.0)
Boxer	1 (0.7)	0 (0.0)	1 (5.0)	0 (0.0)	0 (0.0)	0 (0.0)
Cirneco dell’Etna	32 (22.5)	5 (22.7)	7 (35.0)	5 (35.7)	4 (15.4)	12 (36.4)
Cocker spaniel	2 (1.4)	0 (0.0)	0 (0.0)	0 (0.0)	0 (0.0)	0 (0.0)
Golden retriever	1 (0.7)	0 (0.0)	0 (0.0)	0 (0.0)	0 (0.0)	0 (0.0)
Labrador	8 (5.6)	2 (9.1)	5 (25.0)	1 (7.1)	1 (3.8)	4 (12.1)
Mixed breed	85 (59.9)	15 (68.2)	5 (25.0)	7 (50.0)	17 (65.4)	15 (45.4)
German shepherd	4 (2.8)	0 (0.0)	1 (5.0)	1 (7.1)	2 (7.7)	1 (3.0)
English setter	3 (2.1)	0 (0.0)	0 (0.0)	0 (0.0)	0 (0.0)	0 (0.0)
Spinone italiano	3 (2.1)	0 (0.0)	1 (5.0)	0 (0.0)	1 (3.8)	1 (3.0)
**Reported symptoms**						
Asymptomatic	93 (65.5)	12 (54.5)	11 (55.0)	8 (57.1)	11 (42.3)	18 (54.6)
Symptomatic	49 (34.5)	10 (45.5)	9 (45.0)	6 (42.9)	15 (57.7)	15 (45.4)
***n* symptoms** (min–max)	0–6	0–6	0–5	0–4	0–6	0–5
Symptoms *						
Adenomegalia	20 (40.8)	7 (70.0)	5 (55.5)	2 (33.3)	11 (73.3)	11 (73.3)
Anemia	7 (14.2)	3 (30.0)	0 (0.0)	2 (33.3)	5 (33.3)	4 (26.7)
Anorexia	5 (10.2)	3 (30.0)	0 (0.0)	1 (16.7)	2 (13.3)	1 (6.7)
Cutaneous symptoms	25 (51.0)	6 (60.0)	4 (44.4)	4 (66.7)	15 (100.0)	9 (60.0)
Depression	14 (28.5)	6 (60.0)	2 (22.2)	4 (66.7)	6 (40.0)	6 (40.0)
Diarrhea	4 (8.2)	2 (20.0)	2 (22.2)	1 (16.7)	0 (0.0)	3 (20)
Difficulties with walking	18 (36.7)	3 (30.0)	7 (77.7)	1 (16.7)	6 (40.0)	6 (40.0)
Disorexia	8 (16.3)	5 (50.0)	1 (11.1)	4 (66.7)	2 (13.3)	4 (26.7)
Dyspnea	3 (6.1)	0 (0.0)	1 (11.1)	1 (16.7)	1 (6.7)	1 (6.7)
Edema	15 (30.6)	3 (30.0)	5 (55.5)	3 (50.0)	5 (33.3)	7 (46.7)
Emorragia	5 (10.2)	0 (0.0)	2 (22.2)	0 (0.0)	0 (0.0)	2 (13.3)
Hepatosplenomegaly	1 (2.1)	0 (0.0)	0 (0.0)	0 (0.0)	1 (6.7)	1 (6.7)
Lameness	14 (28.5)	4 (40.0)	5 (55.5)	1 (16.7)	6 (40.0)	7 (46.7)
Low performance	11 (22.4)	2 (18.2)	3 (33.3)	2 (33.3)	4 (26.7)	4 (26.7)
Tenderness	17 (34.6)	2 (18.2)	8 (8.9)	1 (16.7)	6 (40.0)	6 (40.0)
Vomiting	8 (16.3)	3 (30.0)	2 (22.2)	1 (16.7)	1 (6.7)	3 (20)
Weight loss	33 (67.3)	9 (81.8)	5 (55.5)	5 (83.3)	13 (86.7)	13 (86.7)
**Antiparasitic treatmens**						
Yes	50 (35.2)	4 (18.2)	9 (45.0)	4 (28.6)	6 (23.1)	10 (30.3)
Not	92 (64.8)	18 (81.8)	11 (55.0)	10 (71.4)	20 (76.9)	23 (69.7)
**Recovery in a doghouse**						
Yes	33 (23.2)	4 (18.2)	7 (35.0)	1 (7.1)	2 (7.8)	7 (21.2)
Not	109 (76.8)	18 (81.8)	13 (65.0)	13 (92.9)	24 (92.2)	26 (78.8)
**Arising from kennel**						
Yes	94 (66.2)	18 (81.8)	11 (55.0)	11 (78.6)	20 (76.9)	23 (69.7)
Not	48 (33.8)	4 (18.2)	9 (45.0)	3 (21.4)	6 (23.1)	10 (30.3)
**Presence of ticks**						
Yes	41 (28.9)	10 (45.5)	10 (50.0)	5 (35.7)	8 (30.8)	14 (42.4)
Not	101 (71.1)	12 (54.5)	10 (50.0)	9 (64.3)	18 (69.2)	19 (57.6)

^¥^ The total number of positive dogs differs from the total number of positive dogs to each pathogen due to the presence of coinfections. * Percentage is calculated as the ratio between dogs with specific symptoms over the total number of symptomatic dogs; the sum of frequencies differs from 100% because more than one symptom could be reported for each dog.

**Table 2:** Frequency of pathogen coinfections. Data are presented as frequencies and percentage.

Pathogen’ Coinfections	Frequency (%)
*A. phagocytophilum*	6 (8.8)
*B. henselae*	10 (14.7)
*E. canis*	2 (2.9)
*L. infantum*	11 (16.2)
*R. rickettsii*	8 (11.8)
*A. phagocytophilum/L. infantum*	3 (4.4)
*A. phagocytophilum/R. rickettsii*	3 (4.4)
*B. henselae/L. infantum*	1 (1.5)
*B. henselae/R. rickettsii*	3 (4.4)
*E. canis/L. infantum*	1 (1.5)
*E. canis/R. rickettsii*	3 (4.4)
*L. infantum/R. rickettsii*	4 (5.9)
*A. phagocytophilum/B. henselae/E.canis*	1 (1.5)
*A. phagocytophilum/B. henselae/R. rickettsii*	1 (1.5)
*A. phagocytophilum/E.canis/R. rickettsii*	5 (7.4)
*A. phagocytophilum/L. infantum/R. rickettsii*	1 (1.5)
*B. henselae/L. infantum/R. rickettsii*	3 (4.4)
*A. phagocytophilum/B. henselae/L. infantum/R. rickettsii*	1 (1.5)
*A. phagocytophilum/E.canis/L. infantum/R. rickettsii*	1 (1.5)

**Table 3:** Model summary resulting from the MCA.

Dimension	Cronbach’s Alpha [95% CI]	Variance Accounted for Total (Eigenvalue)	% of Variance
1	0.811 [0.705–0.926]	0.296	29.61
2	0.788 [0.583–0.852]	0.143	14.30
3	0.696 [0.665–0.735]	0.114	11.41
Total		0.553	55.32

**Table 4:** MCA dimension discrimination measures.

	MCA Dimension			Mean
	1	2	3	
*A. phagocytophilum*	3.42	0.20	0.00	1.20
*B. henselae*	0.58	4.54	0.07	1.73
*E. canis*	1.64	0.77	0.01	0.80
*L. infantum*	3.62	0.50	4.11	2.74
*R. rickettsii*	3.12	0.50	0.48	1.36
Anemia	4.85	1.51	0.09	2.15
Adenomegalia	7.49	3.68	0.01	3.72
Edema	3.89	1.18	2.60	2.55
Dyspnea	2.18	1.54	1.04	1.58
Depression	2.07	10.32	1.72	4.70
Tenderness	1.32	12.84	1.78	5.31
Difficulties with walking	1.64	10.68	0.90	4.40
Vomiting	4.99	0.22	13.58	6.26
Cutaneous symptoms	2.78	0.31	6.07	3.05
Diarrhea	2.82	0.23	10.97	4.67
Lameness	3.49	10.44	0.36	4.76
Weight loss	7.98	1.00	0.07	2.68
Disorexia	8.09	3.39	4.55	5.34
Anorexia	6.21	2.35	7.55	5.37
Presence of ticks	0.23	0.61	0.94	0.59
Antiparasitic treatments	0.93	6.68	7.74	5.12
Recovery in a doghouse	1.06	6.78	10.19	6.01
Arising from kennel	0.43	3.68	4.07	2.73
Presence of coinfection	4.08	2.53	0.49	2.36
Gender = Female	0.00	1.62	0.46	0.69
Gender = Male	0.00	1.45	0.41	0.62
Age class = adult	0.07	0.36	0.83	0.42
Age class = old	0.26	0.41	0.95	0.54
Age class = young	0.09	0.29	0.66	0.35

## Data Availability

Data are contained within the article.

## References

[1] Mendoza-Roldan J.A., Benelli G., Bezerra-Santos M.A., Nguyen V.L., Conte G., Iatta R., Furlanello T., Otranto D. (2021). Seropositivity to canine tick-borne pathogens in a population of sick dogs in Italy. Parasit. Vectors.

[2] Iatta R., Sazmand A., Nguyen V.L., Nemati F., Ayaz M.M., Bahiraei Z., Zafari S., Giannico A., Greco G., Dantas-Torres F. (2021). Vector-borne pathogens in dogs of different regions of Iran and Pakistan. Parasitol. Res..

[3] Otranto D., Dantas-Torres F. (2010). Canine and feline vector-borne diseases in Italy: Current situation and perspectives. Parasit. Vectors.

[4] Dantas-Torres F., Otranto D. (2014). Dogs, cats, parasites, and humans in Brazil: Opening the black box. Parasit. Vectors.

[5] Otranto D., Dantas-Torres F., Breitschwerdt E.B. (2009). Managing canine vector-borne diseases of zoonotic concern: Part one. Trends Parasitol..

[6] Pérez Pérez P., Rodríguez-Escolar I., Carretón E., Sánchez Agudo J.Á., Lorenzo-Morales J., Montoya-Alonso J.A., Morchón R. (2021). Serological Survey of Canine Vector-Borne Infections in North-Center Spain. Front. Vet. Sci..

[7] Beugnet F., Marie J.L. (2009). Emerging arthropod-borne diseases of companion animals in Europe. Vet. Parasitol..

[8] Angelou A., Gelasakis A.I., Verde N., Pantchev N., Schaper R., Chandrashekar R., Papadopoulos E. (2019). Prevalence and risk factors for selected canine vector-borne diseases in Greece. Parasit. Vectors.

[9] Tadesse H., Grillini M., Simonato G., Mondin A., Dotto G., Frangipane di Regalbono A., Kumsa B., Cassini R., Menandro M.L. (2023). Epidemiological Survey on Tick-Borne Pathogens with Zoonotic Potential in Dog Populations of Southern Ethiopia. Trop. Med. Infect. Dis..

[10] Aoun K., Bouratbine A. (2014). Cutaneous Leishmaniasis in North Africa: A review. Parasite.

[11] Maia C., Cardoso L. (2015). Spread of *Leishmania infantum* in Europe with dog travelling. Vet. Parasitol..

[12] Guccione C., Colomba C., Iaria C., Cascio A. (2023). Rickettsiales in the WHO European Region: An update from a One Health perspective. Parasit. Vectors.

[13] Baneth G. (2011). Perspectives on canine and feline hepatozoonosis. Vet. Parasitol..

[14] Petruccelli A., Ferrara G., Iovane G., Schettini R., Ciarcia R., Caputo V., Pompameo M., Pagnini U., Montagnaro S. (2021). Seroprevalence of *Ehrlichia* spp., *Anaplasma* spp., Borrelia burgdorferi sensu lato, and Dirofilaria immitis in Stray Dogs, from 2016 to 2019, in Southern Italy. Animals.

[15] Giannelli A., Lia R.P., Annoscia G., Buonavoglia C., Lorusso E., Dantas-Torres F., Baneth G., Otranto D. (2017). Rhipicephalus turanicus, a new vector of Hepatozoon canis. Parasitology.

[16] Movilla R., García C., Siebert S., Roura X. (2016). Countrywide serological evaluation of canine prevalence for *Anaplasma* spp., *Borrelia burgdorferi* (*sensu lato*), *Dirofilaria immitis* and *Ehrlichia canis* in Mexico. Parasit. Vectors.

[17] Carrade D., Foley J., Borjesson D., Sykes J. (2009). Canine granulocytic anaplasmosis: A review. J. Vet. Intern. Med..

[18] Evason M., Stull J.W., Pearl D.L., Peregrine A.S., Jardine C., Buch J.S., Lailer Z., O’Connor T., Chandrashekar R., Weese J.S. (2019). Prevalence of Borrelia burgdorferi, Anaplasma spp., Ehrlichia spp. and Dirofilaria immitis in Canadian dogs, 2008 to 2015: A repeat cross-sectional study. Parasit. Vectors.

[19] Stich R.W., Schaefer J.J., Bremer W.G., Needham G.R., Jittapalapong S. (2008). Host surveys, ixodid tick biology and transmission scenarios as related to the tick-borne pathogen, Ehrlichia canis. Vet. Parasitol..

[20] Bellato A., Pintore M.D., Catelan D., Pautasso A., Torina A., Rizzo F., Mandola M.L., Mannelli A., Casalone C., Tomassone L. (2021). Risk of tick-borne zoonoses in urban green areas: A case study from Turin, northwestern Italy. Urban For. Urban Green..

[21] Chisu V., Foxi C., Mannu R., Satta G., Masala G. (2018). A five-year survey of tick species and identification of tick-borne bacteria in Sardinia, Italy. Ticks Tick Borne Dis..

[22] Tamponi C., Scarpa F., Carta S., Knoll S., Sanna D., Gai C., Pipia A.P., Dessì G., Casu M., Varcasia A. (2021). Seroprevalence and risk factors associated with Leishmania infantum in dogs in Sardinia (Italy), an endemic island for leishmaniasis. Parasitol. Res..

[23] Carta S., Sanna D., Scarpa F., Varcasia A., Cavallo L., Meloni M.P., Tamponi C., Cabras P.A., Dessi G., Casu M. (2020). Species diversity and molecular insights into phlebotomine sand flies in Sardinia (Italy)—An endemic region for leishmaniasis. Parasitol. Res..

[24] Cocco R., Sanna G., Cillara M.G., Tola S., Ximenes L., Pinnaparpaglia M.L., Masala G. (2003). Ehrlichiosis and rickettsiosis in a canine population of Northern Sardinia. Ann. N. Y. Acad. Sci..

[25] Masu G., Sechi S., Cocco R., Chisu V., Tanda A., Lollai S., Masala G. (2012). Validation of a serological test for the diagnosis of canine rickettsial disease. Ticks Tick Borne Dis..

[26] Le Roux B., Rouanet H. (2004). Geometric Data Analysis, From Correspondence Analysis to Structured Data Analysis.

[27] Ayele D., Zewotir T., Mwambi H. (2014). Multiple correspondence analysis as a tool for analysis of a large health surveys in African settings. Afr. Health Sci..

[28] Sourial N., Wolfson C., Zhu B., Quail J., Fletcher J., Karunananthan S. (2010). Cor-respondence analysis is a useful tool to uncover the relationships among categorical variables. J. Clin. Epidemiol..

[29] Lê S., Josse J., Husson F. (2008). FactoMineR: An R package for multivariate analysis. J. Stat. Softw..

[30] Hair J.F., Tatham R.L., Anderson R.E., Black W. (1998). Multivariate Data Analysis.

[31] Vaissie P., Monge A., Husson F. (2023). Perform Factorial Analysis from ‘FactoMineR’ with a Shiny Application.

[32] Solano-Gallego L., Caprì A., Pennisi M.G., Caldin M., Furlanello T., Trotta M. (2015). Acute febrile illness is associated with *Rickettsia* spp. infection in dogs. Parasit. Vectors.

[33] Stewart A.G., Stewart A.G.A. (2021). An Update on the Laboratory Diagnosis of *Rickettsia* spp. Infection. Pathogens.

[34] Santibáñez S., Portillo A., Ibarra V., Santibáñez P., Metola L., García-García C., Palomar A.M., Cervera-Acedo C., Alba J., Blanco J.R. (2022). Epidemiological, Clinical, and Microbiological Characteristics in a Large Series of Patients Affected by *Dermacentor*-Borne-Necrosis-Erythema-Lymphadenopathy from a Unique Centre from Spain. Pathogens.

[35] Chisu V., Zobba R., Foxi C., Pisu D., Masala G., Alberti A. (2016). Molecular detection and groEL typing of *Rickettsia aeschlimannii* in Sardinian ticks. Parasitol. Res..

[36] Solano-Gallego L., Miró G., Koutinas A., Cardoso L., Pennisi M.G., Ferrer L., Bourdeau P., Oliva G., Baneth G. (2011). LeishVet guidelines for the practical management of canine leishmaniosis. Parasit. Vectors.

[37] Madeddu G., Fiori M.L., Ena P., Riu F., Lovigu C., Nunnari G., Bagella P., Maida I., Babudieri S., Mura M.S. (2014). Mucocutaneous leishmaniasis as presentation of HIV infection in Sardinia, insular Italy. Parasitol. Int..

[38] Berzina I., Krudewig C., Silaghi C., Matise I., Ranka R., Müller N., Welle M. (2014). *Anaplasma phagocytophilum* DNA amplified from lesional skin of seropositive dogs. Ticks Tick Borne Dis..

[39] Martinescu G.V., Ivănescu L., Ștefănescu R., Andronic L., Mătiuț S., Mîndru R., Solcan G., Miron L. (2023). Strategies for the Diagnosis of Granulocytic Anaplasmosis in Two Naturally Infected Dogs. Animals.

[40] Masala G., Chisu V., Foxi C., Socolovschi C., Raoult D., Parola P. (2012). First detection of *Ehrlichia canis* in *Rhipicephalus bursa* ticks in Sardinia, Italy. Ticks Tick Borne Dis..

[41] Aziz M.U., Hussain S., Song B., Ghauri H.N., Zeb J., Sparagano O.A. (2022). Ehrlichiosis in Dogs: A Comprehensive Review about the Pathogen and Its Vectors with Emphasis on South and East Asian Countries. Vet. Sci..

[42] Zobba R., Chessa G., Mastrandrea S., PinnaParpaglia M.L., Patta C., Masala G. (2009). Serological and molecular detection of Bartonella spp. in humans, cats and dogs from northern Sardinia, Italy. Clin. Microbiol. Infect..

[43] Chomel B.B., Boulouis H.J., Maruyama S., Breitschwerdt E.B. (2006). *Bartonella* spp. in pets and effect on human health. Emerg Infect. Dis..

[44] Easley F., Taylor L., B Breitschwerdt E. (2021). Suspected *Bartonella* osteomyelitis in a dog. Clin. Case Rep..

